# Structural Plasticity of Dendritic Spines Requires GSK3α and GSK3β

**DOI:** 10.1371/journal.pone.0134018

**Published:** 2015-07-24

**Authors:** Iwona A. Cymerman, Agata Gozdz, Malgorzata Urbanska, Jacek Milek, Magdalena Dziembowska, Jacek Jaworski

**Affiliations:** 1 The International Institute of Molecular and Cell Biology, Warsaw, Poland; 2 Laboratory of Neurobiology, The Nencki Institute, Warsaw, Poland; 3 Centre of New Technologies, University of Warsaw, Warsaw, Poland; University of Nebraska Medical Center, UNITED STATES

## Abstract

Although memories appear to be elusive phenomena, they are stored in the network of physical connections between neurons. Dendritic spines, which are actin-rich dendritic protrusions, serve as the contact points between networked neurons. The spines’ shape contributes to the strength of signal transmission. To acquire and store information, dendritic spines must remain plastic, i.e., able to respond to signals, by changing their shape. We asked whether glycogen synthase kinase (GSK) 3α and GSK3β, which are implicated in diseases with neuropsychiatric symptoms, such as Alzheimer's disease, bipolar disease and schizophrenia, play a role in a spine structural plasticity. We used Latrunculin B, an actin polymerization inhibitor, and chemically induced Long-Term Depression to trigger fast spine shape remodeling in cultured hippocampal neurons. Spine shrinkage induced by either stimulus required GSK3α activity. GSK3β activity was only important for spine structural changes after treatment with Latrunculin B. Our results indicate that GSK3α is an essential component for short-term spine structural plasticity. This specific function should be considered in future studies of neurodegenerative diseases and neuropsychiatric conditions that originate from suboptimal levels of GSK3α/β activity.

## Introduction

Dendritic spines are small protrusions that serve as postsynaptic biochemical compartments specialized in receiving excitatory input. The spines capability to undergo changes in morphology and synapse conductance is the basis for long-term synaptic plasticity [1,2], the process that is thought to underlie learning and memory. The morphological and functional heterogeneity of spines results from the constant and mutual adjustment of spine structure to function: different modalities of synaptic activity, which lead to changes in synaptic transmission, may also affect spine shape [1,2]. Spine shape itself significantly contributes to activation of the synapse hosted by the spine. For example, reduced synaptic responsiveness is followed by spine shrinkage in NMDA receptor-dependent Long-Term Depression (NMDAR-LTD) [3]. However, changes in synapse strength and spine structure can be “unsynchronized” (e.g., in NMDAR-LTD upon inhibition of phosphatases PP1/2A) [3]. This suggests that NMDAR-LTD-dependent synaptic and structural responses share their initial biochemical steps and subsequently diverge. Regardless of the trigger, any spine structural changes have to involve the F-actin cytoskeleton, which is the scaffold of spine structure [4].

GSK3α and GSKβ are homeostatic kinases that act at the crossroads of numerous signaling pathways [5]. GSK3α and GSK3β function properly only within a defined physiological range. Therefore, either over-activation or insufficient activity of GSK3s profoundly affects nervous system development and function [6]. GSK3β knock-out (KO) mice die at an embryonic stage [7]. GSK3α KO mice survive [8], although they suffer from deregulated synaptic transmission and cognitive impairment [9]. Long-term, conditional-KO of neuronal GSK3β causes the loss of persistent spines and reduces the stability of newly formed spines [10]. Although majority of studies on GSK3 in the nervous system physiology and pathology focused on effects of chronic long term changes of GSK3 activity, the evidence exist for physiological relevance of GSK3 activity fluctuation in much shorter time scale. For example, at the electrophysiological level, GSK3α and GSK3β are inhibited by Long-Term Potentiation [11]. What is more, among 58 serine/threonine kinases tested, GSK3α and GSKβ are only kinases required for LTD maintenance [12].

Despite such strong evidence for crucial role of GSK3 in synaptic plasticity, there have been no reports of changes in GSK3-dependent short-term structural plasticity accompanying changes in synaptic conductance, despite the fact that some GSK3 substrates are related to both synaptic and structural plasticity e.g., NMDAR [13], PSD-95 [14], AMPAR [15]. To verify whether GSK3α/β activity influences short-term spine structural changes, we used live time-lapse imaging. Spine structure was monitored after the addition of Latrunculin B (LatrB, a drug that prevents F-actin assembly) or chemical LTD (chLTD) treatments and prior to the chemical inhibition of GSK3α/β (GSK3α/β inhibitors Ch98 and BIO) or RNAi-mediated depletion (by individual/specific silencing of GSK3α and GSKβ).

## Materials and Methods

### DNA constructs and antibodies

pβactin-EGFP plasmid encoding EGFP under control of β-actin promoter was described previously [16]. pSUPER^GFP^ [17] were the backbone for cloning shRNA sequences. shGSK3α#9-#11, shGSK3β#12-#15 and shGSK3α/β#17 were designed with the siRNA Selection Program [18] against murine GSK3α and GSK3β mRNA sequences (see Table 1). shGSK3α# 8 was previously described [19]. pSUPER^GFP^ plasmids carrying scrambled shRNAs were designed based on the original shRNA sequences using the on-line GeneScript tool. Scrambled shRNA sequences (see Table 1) were used as a query in BLASTn [20] searches to eliminate sequences that might target transcription of other murine mRNAs. To minimize side effects, neurons were transfected with a mixture of shRNAs or scrambled shRNAs as shown in the Table 1. Antibodies used in the study are presented in Table 2.

**Table 1:** shRNA and scramble shRNA designed and used in the study.

	shRNA name	shRNA target site	scramble RNA sequence
	GSK3α murine mRNA (NM_001031667.1)		
Mixture α	pSUPER^GFP^- shGSK3α#8	574–594	ATCGTAATTCGCGTTACCGAG
	pSUPER^GFP^- shGSK3α#9	540–558	AGTCAACCGTTATTGACGC
	pSUPER^GFP^- shGSK3α#10	2073–2091	ATCTCGCTACATAACACTC
	pSUPER^GFP^- shGSK3α#11	1014–1032	GACCTCCTACGTACTTCTA
	GSK3β murine mRNA (AF156099)		
Mixture β	pSUPER^GFP^-shGSK3β#12	980–998	ACAACCTAGTACGAGCAAC
	pSUPER^GFP^-shGSK3β#13	619–637	AGATTCGATCCGCCTTATC
	pSUPER^GFP^-shGSK3β#14	541–559	ACCTGGTTACTCGTGTAGC
	pSUPER^GFP^-shGSK3β#15	1155–1173	ACGACGGTATCCTACCGGT
	GSK3α (NM_001031667.1) and GSK3β (AF156099) murine mRNA		
	pSUPER^GFP^- shGSK3α/β#17	GSK3α: 662-680GSK3β: 448–466	v1:ATCGACATGCGAAGCCGCAv2:ACTAACGCGAGAGTCCGCA

**Table 2:** Antibodies used in the study.

Primary antibody	Manufacturer Cat. No; LOT; dilution	Secondary antibody
Odyssey Imaging System		
anti-rabbit phospho-GluA1 (GluR1) Ser845	Millipore #04–1073; 2056718; 1:1000	anti-rabbit IgG, IRDye 800CW; Li-Cor #926–68023; 1:10000
anti-rabbit phospho-GSK3α/β Ser21/9	Cell Signaling #9331; 13; 1:2000	anti-rabbit IgG, IRDye 800CW; Li-Cor #926–68023; 1:10000
anti-rabbit GAPDH	Synaptic Systems #247002; 247002g/x; 1:1000	anti-rabbit IgG, IRDye 800CW; Li-Cor #926–68023; 1:10000
anti-mouse GSK3α/β	Life Technologies #44610; 73250821A; 1:3000	anti-mouse IgG, IRDye 680LT; Li-Cor #926–32212; 1:10000
anti-mouse α-tubulin	Sigma-Aldrich #T5168; 072M4809; 1:1000	anti-mouse IgG, IRDye 680LT; Li-Cor #926–32212; 1:10000
Enhanced chemiluminescent detection (ECL)		
anti-rabbit phospho-Glycogen Synthase Ser641	Cell Signaling #3891; 2; 1:1000	anti-rabbit IgG, HRP-linked; Cell Signaling #7074; 1:20000
Immunofluorescence		
anti-rabbit GSK3α	Cell Signaling #4818; 1; 1:200	anti-rabbit IgG Alexa Fluor 647; A-21443; 1:200
anti-mouse GSK3β	BD #610201; 60272; 1:200	anti-mouse IgG Alexa Fluor 555; A-21425; 1:200

### Primary hippocampal neuron cultures

Neurons for *in vitro* cultures were obtained from embryonic murine brains: female mice were euthanized by cervical dislocation and isolated embryos (E17) were immediately decapitated., Neurons were grown in Neurobasal medium (Life Technologies) supplemented with B27 (Life Technologies), glutamine, glutamate and Penicillin-Streptomycin (all from Sigma-Aldrich). Neurons were transfected with Lipofectamine 2000 (Life Technologies) at DIV16 [17]. Dendritic spine morphology was visualized by EGFP expression. To silence the expression of GSK3α and GSK3β, neurons were transfected with pSUPER^GFP^-shRNA constructs (Table 1). Forty-eight hours post-transfection, neurons were imaged live or fixed (4% PFA with 4% sucrose in PBS) for immunofluorescence labeling according to the manufacturer’s recommendations.

### Synaptoneurosome preparation

Synaptoneurosomes were prepared from 1–2-month-old, wild-type mice as described previously [21]. Briefly, hippocampi from one mouse were dissected and homogenized at 4°C in 1 ml of homogenization buffer (125 mM NaCl, 1.2 mM MgSO_4_, 2.5 mM CaCl_2_, 1.53 mM KH_2_PO_4_, 212.7 mM glucose, 4 mM NaHCO_3_ at pH 7.4), set with carbogen, supplemented with Protease Inhibitor Cocktail (Sigma-Aldrich) and 100 U/ml mammalian placental RNase inhibitor (Thermo Scientific). The final volume of the homogenate was adjusted to 8 ml with homogenization buffer. Homogenate samples were consecutively passed through series of nylon mesh filters of 100, 60, 30 and 10 μm (Millipore) then centrifuged at 1000 x g for 15 min. The pellets containing synaptoneurosome preparations were washed in homogenization buffer, centrifuged as before and resuspended in homogenization buffer.

### GSK3α/β chemical inhibition

GSK3α/β kinase activity was blocked by the specific inhibitors Chiron 98014 (Ch98; 0.5 μM; Axon Medchem, The Netherlands) and BIO (1 μM; Tocris, Bristol, UK) dissolved in DMSO. Drugs were added to the growth media 60 min. before further treatments.

### Latrunculin B treatment

Latrunculin B (0.5 μM, Calbiochem) was dissolved in DMSO and administered for 20 min. Next, the drug was removed and the treated cells were returned to conditioned growth medium.

### Chemical LTD

To induce chLTD neurons were washed 3 times with Extracellular fluid (ECF: 130 mM NaCl, 2.5 mM KCl, 2.2 mM CaCl_2_, 1.5 mM MgCl_2_, 10 mM Hepes, 10 mM D-Glucose and Osm 290 at pH 7.3–7.4) supplemented with 1 μM TTX (Alomone Labs) and then incubated for 5 min with chLTD solution (ECF supplemented with 1 μM TTX, 20 μM NMDA [Sigma-Aldrich] and 20 μM glycin [Sigma-Aldrich]). After treatment, cells were washed 3 times with ECF and returned to conditioned medium with or without inhibitors, depending on the experiment. To induce chLTD in synaptoneurosomes, freshly isolated synaptoneurosomes were incubated with 1 μM TTX for 5 min. NMDA and glycine were added to a final concentration of 20 μM for a 5 min. incubation. Finally, synaptoneurosomes were washed with homogenization buffer and incubated for 5, 10 and 15 minutes.

### Imaging and image analysis

Time-lapse images of live dendritic spines and images of fixed, immunofluorescently stained neurons were recorded with a ZEISS 710 NLO confocal system equipped with a 40x/1.1 W Corr objective for water immersion and 40x1.3 objective for oil-immersion. Images were analyzed using MetaMorph image analysis software (Universal Imaging). The only criterion for spine to be included in the analysis was its presence during the entire experiment and therefore all spines which met this criterion were included in the analysis regardless the fact if their shape changed or not. Length and width of each individual spine were monitored overtime. The mean lengths and widths of spines of the particular neuron in the given time point were compared to the initial means of spine length and width and expressed as % change. Temporal changes in the spine length/width ratio (l/w) were compared and presented as % cumulative change in the l/w for three time points: initial observation, treatment and recovery. For quantification of the effects of shRNAs on endogenous proteins levels in transfected neurons, cell images were captured with the sequential scanning function using identical settings across experimental groups. The intensity values of GSK3α or GSK3β staining in transfected cells were measured from the cell soma of a single z-stack and then normalized to the average staining intensity of three neighboring, non-transfected neurons.

### Statistical analysis

All data were obtained from at least three biological replicates. The results were analyzed with GraphPad Prism software (GraphPad, La Jolla, CA). The Mann-Whitney U test, Kruskal-Wallis ANOVA or a one-sample t-test were used to verify statistical significance.

### Ethics statement

Procedures to obtain starting material for *in vitro* cultures from embryonic (E17) murine brains used for the studies described herein were approved by the First Local Ethics Committee in Warsaw (Decisions No 951/2009 & 198/2011) and are in compliance with the European Community Council Directive (86/609/EEC).

## Results

### GSK3α/β inhibition prevents spine structural changes induced by F-actin depolymerization and chemical LTD

**Fig 1:**
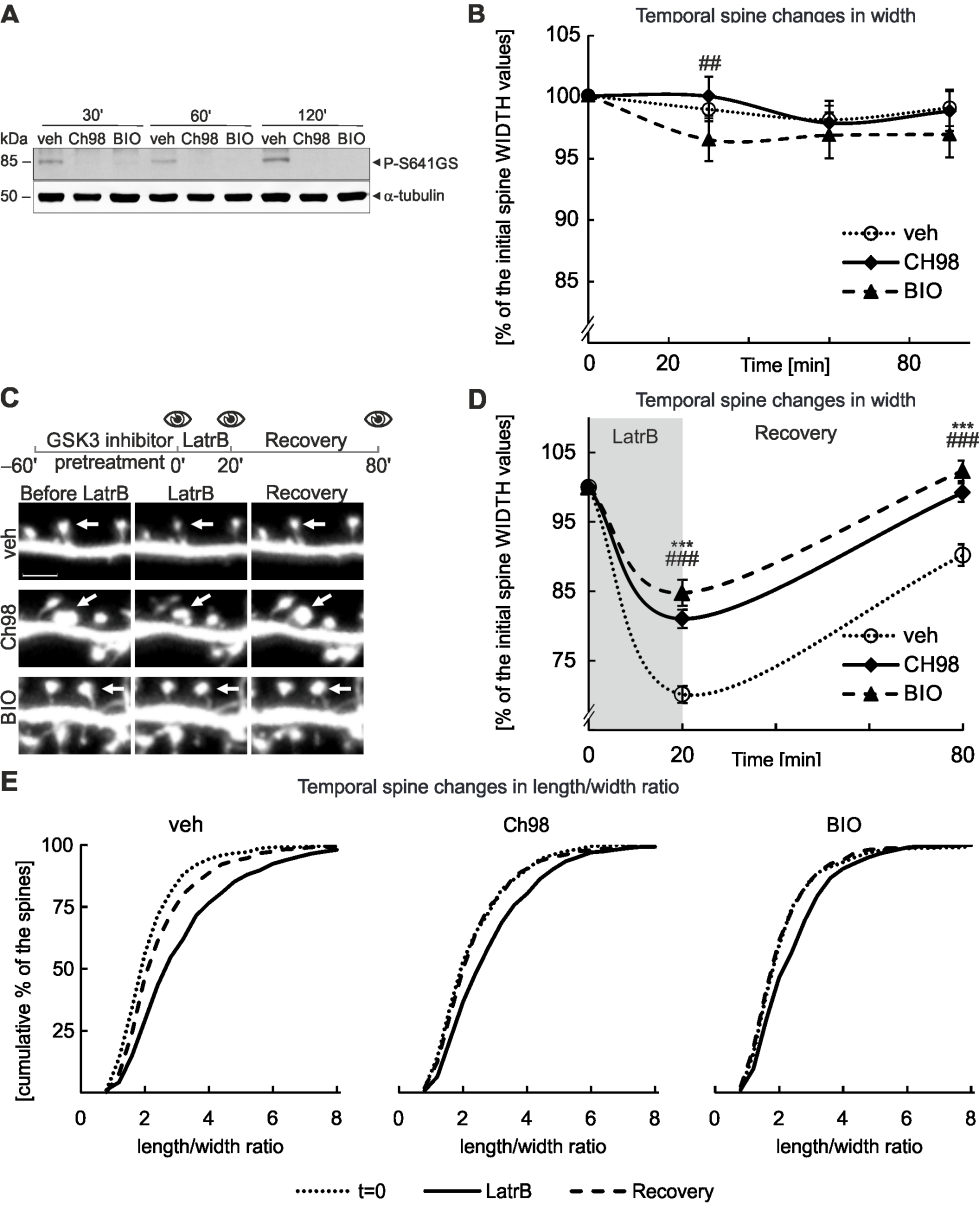
Inhibition of GSK3α/β activity in cultured neurons treated with LatrB hinders fast spine structural changes. A. Efficiency of GSK3α/β chemical inhibition. The level of phosphorylation for glycogen synthase Ser 641 at time points after GSK3α/β inhibition with Ch98 and BIO. Lysates from hippocampal neurons on DIV18. Tubulin was the loading control. B. Pharmacological inhibition of GSK3α/β does not affect basal fluctuations of dendritic spine morphology. Experimental outline with 4 time points for microscopy and quantitative analysis of spine width; ## indicates *p*<0.01 for measurements of spines after GSK3α/β inhibition with BIO compared with control values at the corresponding time point. For number of counted spines refer to Table 3. Data are presented as the mean spine width per cell ± s.e.m. The curve between time points is extrapolated. C. Experimental outline with 3 time points for microscopy: baseline, LatrB treatment, end of recovery period. Representative micrographs of DIV18 cultured murine hippocampal neurons. Scale bar = 2.5 μm. D. Quantitative analysis of spine shape changes; *** and ### indicates *p*<0.001 for measurements of spines after GSK3α/β inhibition by Ch98 and BIO when compared to control values at the corresponding time points. For number of counted spines refer to Table 3. Data are presented as mean spine width per cell ± s.e.m. The curve between time points is extrapolated. E. Spine l/w ratio changes are presented as cumulative histograms of the l/w ratio at 3 time points.

Research on GSK3α/β and spine morphology has typically been focused on long-term, chronic changes in kinase activity. However, the rapid dynamics of spine structural plasticity requires fast and continuous monitoring of spine morphology together with acute manipulations of GSK3α and GSK3β activities. For the short-term inhibition of GSK3 activity, we applied two structurally unrelated GSK3α/β inhibitors: Ch98 and BIO. Thirty minutes after the administration of Ch98 and BIO, phosphorylation levels of glycogen synthase (GS, the substrate for GSK3α/β) were substantially reduced. After 1 hour, phosphorylation was not detectable (Fig 1A), indicating the functional inhibition of GSK3α/β. Although Ch98 and BIO are very potent, showing biochemical changes within 30 minutes of administration (Fig 1A), they did not affect the intrinsic spine structural fluctuations for at least 90 minutes (Fig 1B, S1 Fig), as shown by time-lapse imaging of live, mature (DIV18) hippocampal neurons in primary culture.

**Table 3:** Sample sizes and the standard errors of the mean for experimental groups.

Fig	variant	No of spines	No of cells (*n*)	No of individual cultures	SEM (of given time points)		
					**30’**	**60’**	**90’**
**1B**	veh	436	20	3	0.009	0.015	0.014
	Bio	322	18	3	0.017	0.018	0.018
	Ch98	267	14	2	0.015	0.014	0.016
**4A**	pSuper	436	21	3	0.009	0.014	0.014
	shGSK3α	209	11	2	0.023	0.016	0.020
	skGSK3β	176	10	2	0.022	0.025	0.024
	shGSK3α/β	280	14	2	0.019	0.020	0.019
					**20’**	**80’**	
**1D**	veh	461	19	4	0.012	0.015	-
	Bio	421	19	4	0.018	0.014	-
	Ch98	438	20	4	0.013	0.013	-
**4C**	pSuper	421	19	3	0.011	0.011	-
	shGSK3α	367	16	3	0.027	0.018	-
	skGSK3β	283	14	3	0.028	0.023	-
	shGSK3α/β	307	15	3	0.021	0.015	-
					**15’**	**75’**	
**2D**	veh	602	24	5	0.011	0.014	-
	Bio	437	16	4	0.016	0.009	-
	Ch98	361	15	4	0.020	0.019	-
**5B**	pSuper	435	18	3	0.014	0.022	-
	shGSK3α	364	16	3	0.020	0.019	-
	skGSK3β	367	17	3	0.018	0.028	-
	shGSK3α/β	340	15	3	0.023	0.026	-

We next verified the effect of GSK3α/β inhibition on short-term structural spine changes evoked by the depolymerization of F-actin, a main component of the spine cytoskeleton. The formation of F-actin can be prevented by short-term treatment with LatrB, which reversibly depletes the accessible pool of monomeric actin (Fig 1C). Using live time-lapse imaging, we confirmed that LatrB induces significant changes in the spine shape of mature (DIV18) hippocampal neurons in primary culture: spine width decreased by 30% during incubation with LatrB and returned to 90% of the initial width 1 h after LatrB washout because of F-actin re-polymerization. In contrast, when GSK3α and GSK3β were inhibited by a 1 hour pretreatment with Ch98 or BIO, spine width decreased by 15% during LatrB treatment and was completely restored during the recovery phase (Fig 1C and 1D). No substantial differences in spine length were observed between experimental variants (S1 Fig). Spine functional status depends on and is reflected by its volume [22] that in 2D space is represented by the length and width of the spine. The population of spines exists in the variety of shapes, thus the distribution of length and width is continuous. Therefore to analyze changes in spine shape, which indirectly could reflect functional changes, across the whole analyzed spine population we decided to look at distribution of length to width (l/w) ratio. In neurons with GSK3α/β inhibition, we observed a smaller shift toward greater length/width ratio values compared to the control (Fig 1E), indicating a decreased potential to undergo structural changes. Although LatrB does not reflect any physiological condition, these data already indicate that chemical inhibition of GSK3α/β hinders the short-term structural changes evoked by LatrB-mediated inhibition of F-actin dynamics as well as show the maximal spine shrinkage that can be obtained and imaged in our model system.

**Fig 2:**
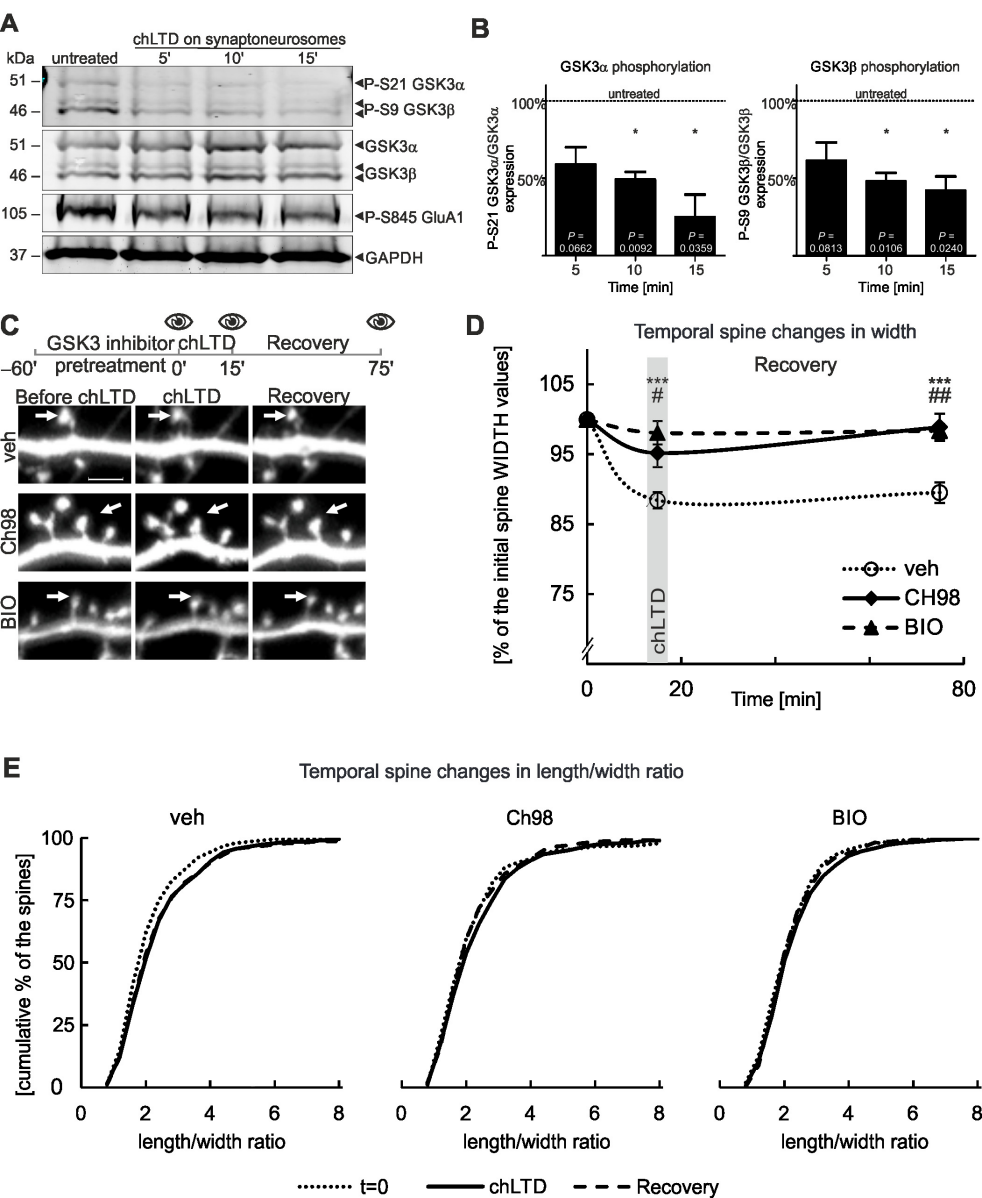
Inhibition of GSK3α/β activity in cultured neurons hinders spine structural plasticity upon chLTD A. chLTD activates GSK3α/β in synaptoneurosomes isolated from murine hippocampi. Representative immunoblots, scanned with a Li-Cor Odyssey imager, for phospho-GSK3α (Ser21)/β (Ser9), total GSK3α/β, phospho-GluA1 (Ser845; LTD control) and GAPDH (loading control). B. Quantitative analysis of phospho-GSK3α/β to the total-GSK3α/β ratio at the time points indicated, expressed as % values of an untreated control. Data (n = 3 experiments) are presented as means ± s.e.m. * indicates a *p*<0.05 vs control. C. Pharmacological inhibition of GSK3α/β affects chemical LTD-induced changes of dendritic spine width. Experimental outline with 3 time points for microscopy: baseline, chLTD induction, end of recovery period. Representative micrographs of DIV18 cultured murine hippocampal neurons. Scale bar = 2.5 μm. D. Quantitative analysis of spine shape changes; *** = *p* < 0.001, # = *p* < 0.05, ## = *p* <0.01 for spine measurements after GSK3α/β inhibition with Ch98 and BIO compared to control values at the corresponding time points. For number of counted spines refer to Table 3. Data are presented as mean spine width per cell ± s.e.m. The curve between time points is extrapolated. E. Spine l/w ratio changes are presented as cumulative histograms of the length/width ratio at 3 time points.

Due to the importance of GSK3α/β in LTD and the known reliance of LTD on F-actin dynamics, we decided to test the hypothesis if upon LTD induction the shift in GSK3α/β activity will be also manifested on the level of spine structural changes. Thus we decided to monitor the effects of GSK3α/β inhibition on spine morphology in combination with NMDA-glycine induced chemical LTD (chLTD). First, we verified that chLTD activates GSK3α/β at the synapse using isolated synaptoneurosomes. As a control, we monitored levels of the GluA1 subunit of the AMPA receptor, which undergoes dephosphorylation at Serine 845 [23] (Fig 2A). The levels of phosphorylated GSK3α and GSK3β, on Ser21 and Ser9 respectively, were decreased by chLTD indicating an increase in activity (Fig 2A and 2B). Using time-lapse live imaging (Fig 2C), we confirmed that chLTD induces significant spine shape changes in mature (DIV18) hippocampal neurons in primary culture. Spine width was decreased by 12% (~ 30% of the maximal spine width decrease that can be induced by LatrB) during chLTD-mediated induction of LTD. This decrease lasted throughout the recovery phase (1h after chLTD washout; Fig 2C and 2D). We did not observe substantial changes of spine length in the presence of chLTD (S1 Fig). In contrast to the control group, when GSK3α and GSK3β were inhibited by a 1 hour pretreatment with Ch98 or BIO, spine width decreased by a maximum of 5% and was restored during the recovery phase of chLTD treatments (Fig 2C and 2D). Morphologically, neurons with inhibited GSK3α/β displayed a smaller shift toward greater l/w ratios compared to the control during chLTD (Fig 2E). The shift at the end of recovery period was maintained in the control group while the l/w ratio was unchanged in neurons with GSK3α/β inhibition, indicating a reduced potential for structural changes. These results show that under basal conditions, inhibition of GSK3α/β prevents structural changes in response to chLTD without influencing spine shape.

### GSK3α and GSKβ play separate roles in spine changes

**Fig 3:**
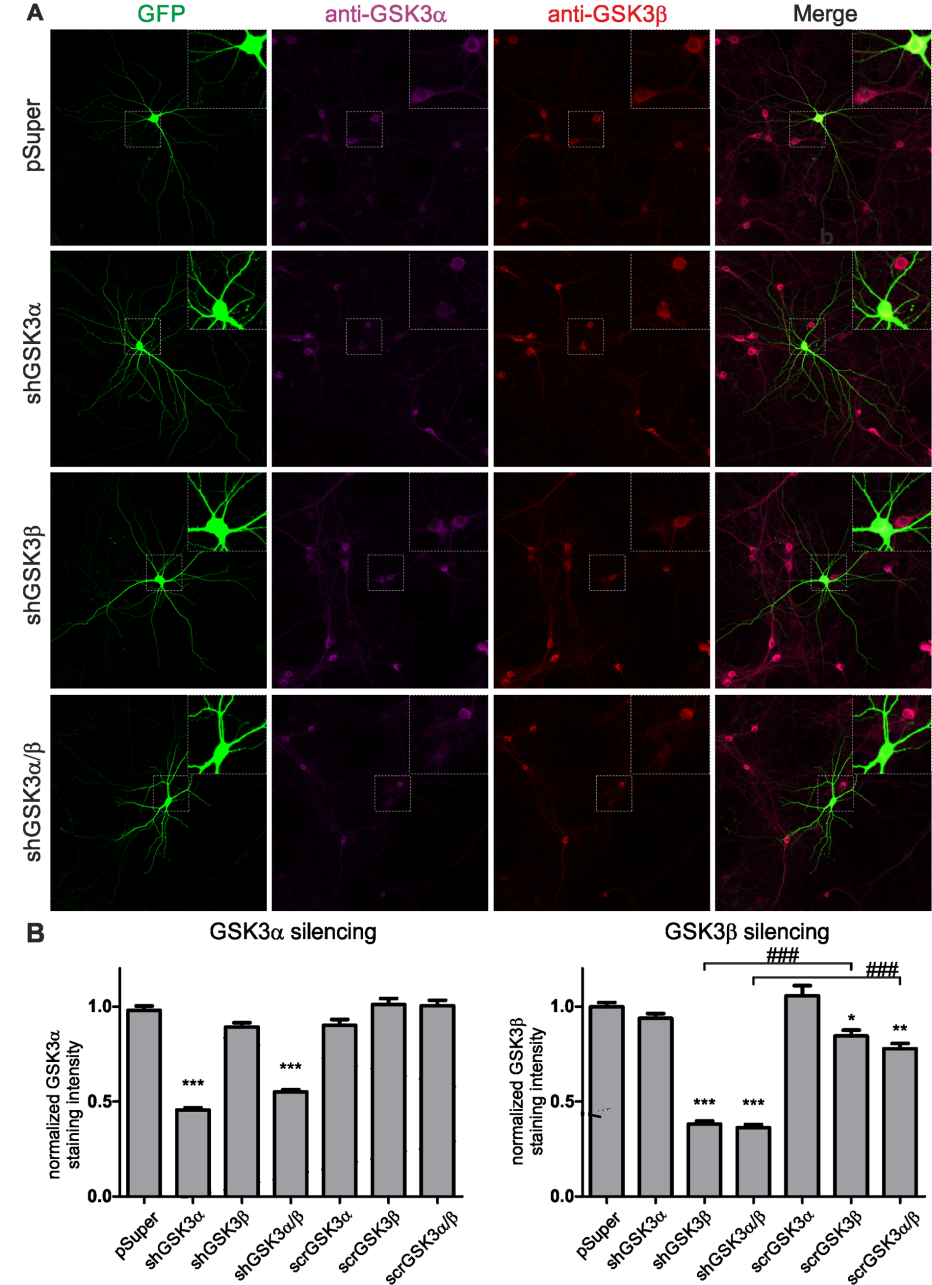
Validation of GSK3 shRNAs and their scrambled counterparts. A. Representative micrographs of neurons transfected with shRNA constructs (as indicated) and immunofluorescently labeled for GSK3α or GSK3β. Scale bar = 50 μm. B. Quantitative analysis of GSK3 silencing; * = *p* < 0.05, ** = *p* <0.01 and *** = *p* < 0.001 for intensity measurements compared to neurons expressing pSuper^GFP^. ### = *p* < 0.001 for experimental variants indicated by brackets. Data are presented as the mean ± s.e.m.

To date, most attention has been focused on GSK3β, yet recent studies clearly point to neuronal functions for GSK3α [9] and distinguish its role in synaptic plasticity from that of GSK3β [24]. Because commercially available GSK3α/β inhibitors do not discriminate between GSK3α and GSK3β, we used molecular tools for selective inhibition. As observed in knock-out mice, the absence of one kinase was compensated for by the up-regulation of the other [25], we used RNAi, for a more acute approach, to selectively silence GSK3α or GSK3β expression in mature hippocampal neuron cultures (Fig 3). GSK3α/β shRNA effectively diminished GSK3α, GSK3β and GSK3α/β levels 48 h post-transfection in cultured neurons (Fig 3A and 3B). We also observed a slight decrease in GSK3β expression upon transfection with scrambled shRNA—scrGSK3β and scrGSK3α/β (Fig 3B) but subsequent functional analysis showed that the decrease was not sufficient to influence spine structural plasticity (see below).

**Fig 4:**
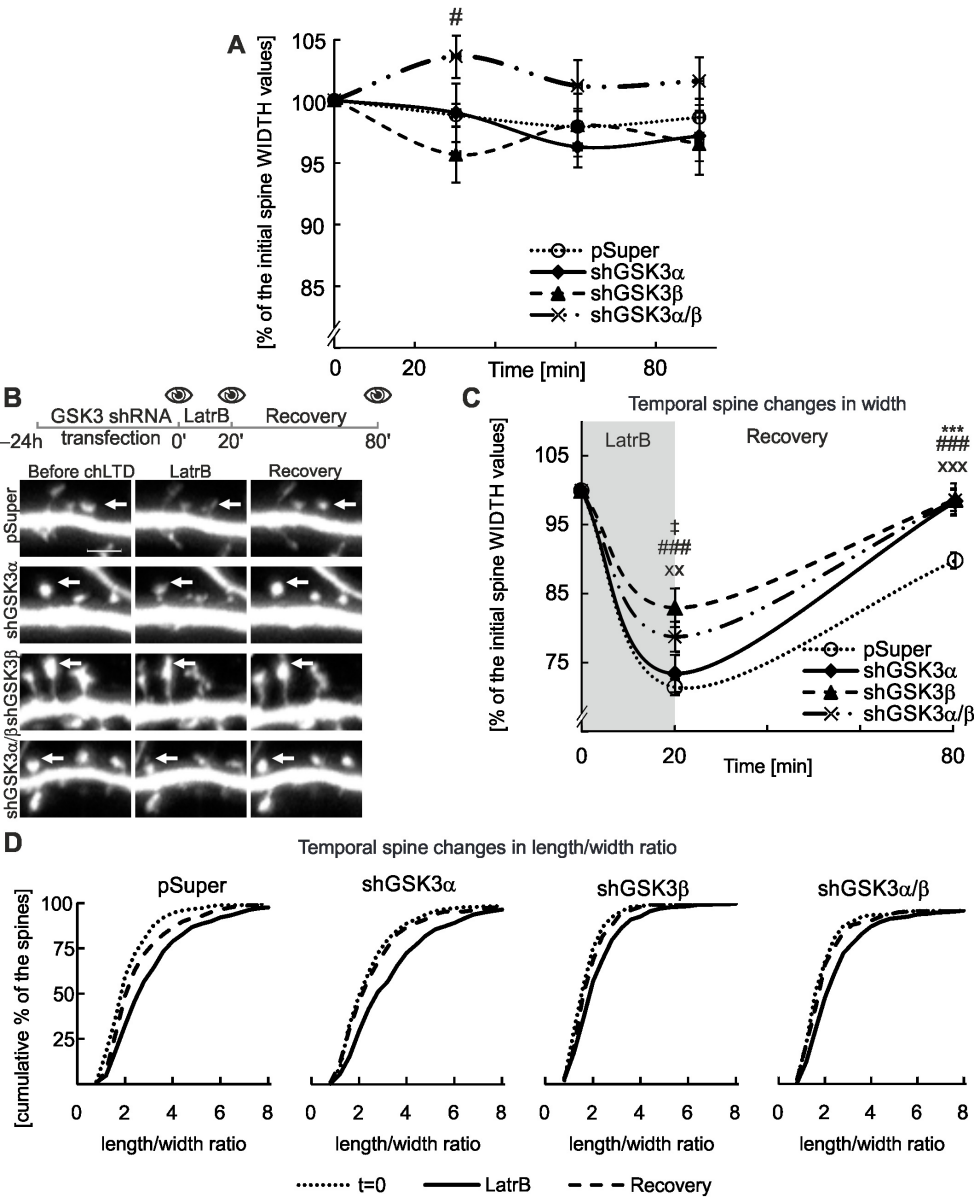
GSK3α and GSK3β knockdown alter LatrB-induced changes to dendritic spine morphology. A. GSK3 α/β knockdown does not affect basal fluctuations of dendritic spine morphology. Experimental outline with 4 time points for microscopy and quantitative analysis of spine shape; # indicates *p*<0.05 for measurements of spines after GSK3β silencing compared to control values at the corresponding time points. For number of counted spines refer to Table 3. Data are presented as the mean spine width per cell ± s.e.m. The curve between time points is extrapolated. B. Experimental outline with 3 time points for microscopy: baseline, LatrB treatment, end of recovery period. Representative micrographs of cultured DIV18 murine hippocampal neurons transfected with shRNA constructs as indicated. Scale bar = 2.5 μm. C. Quantitative analysis of spine shape changes; *** = *p* < 0.001 and xx = *p* < 0.01, xxx = *p* < 0.001 and ### = *p* < 0.001 for spine measurements of shRNA silenced GSK3α, GSK3β and GSK3α/β compared to the control at the corresponding time points. ‡ = *p* < 0.05 difference between shRNAGSK3α and shGSK3β. For number of counted spines refer to Table 3. Data are presented as mean spine width per cell ± s.e.m. The curve between time points is extrapolated. D. Spine l/w ratio changes presented as cumulative histograms of length/width ratio at 3 time points.

Using these tools, we compared the effects of GSK3α versus GSK3β inhibition on basal spine shape fluctuations and on structural spine changes induced by LatrB and chLTD. As with pharmacological inhibition, neither GSK3α nor GSK3β shRNA substantially affected the basal fluctuations of spine width (Fig 4A, S1 Fig,). LatrB induced spine shape changes upon silencing of either kinase. Nevertheless, we observed variations between the effects of GSK3α and GSK3β knockdown (Fig 4B and 4C). Incubation with LatrB reduced spine width by 30% in control conditions and with GSK3α silencing. In cells transfected with either GSK3β or GSK3α/β shRNA, spine width only decreased by 15–20%, similar to the decrease observed after treatment with GSK3α/β inhibitors. During the recovery phase, spines treated with any of the GSK3 shRNA variants fully recovered their initial shapes, regardless of the level of LatrB-induced spine shrinkage (Fig 4B and 4C). In contrast, spines in the control condition only returned to 90% of their initial spine width (Fig 4B and 4C). This difference is also reflected by the l/w ratio (Fig 4D). No significant changes were observed in the spine length (S1 Fig).

**Fig 5:**
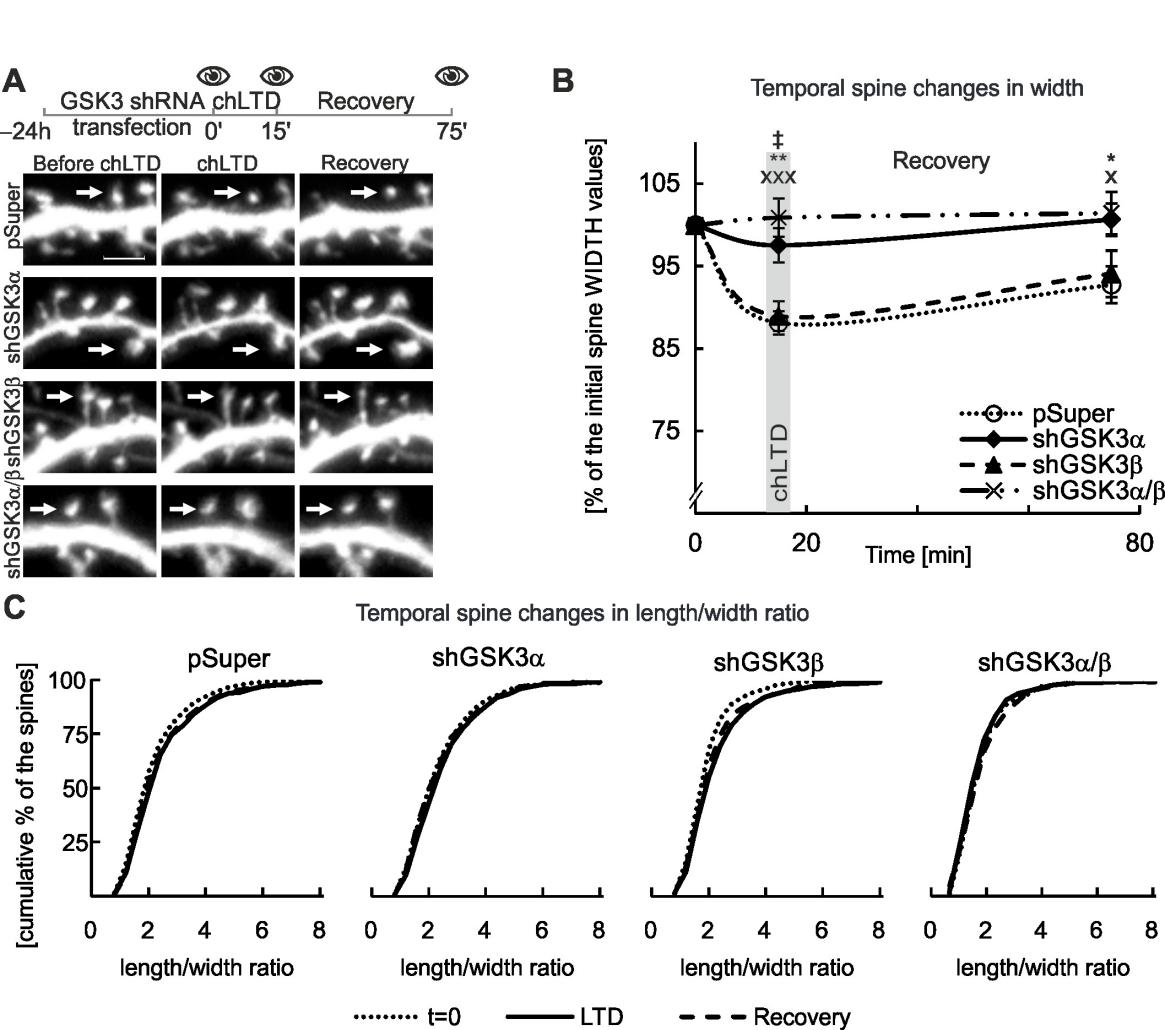
Knockdown of GSK3α but not GSK3β affects chLTD-induced changes to dendritic spine morphology A. Experimental outline with 3 time points for microscopy: baseline, chLTD induction, end of recovery period. Representative micrographs of cultured DIV18 murine hippocampal neurons transfected with shRNA constructs as indicated. Scale bar = 2.5 μm. B. Quantitative analysis of spine shape changes; * = *p* < 0.05, ** = *p* < 0.01 and x = *p* <0.05 and xxx = *p* < 0.001 for measurements of spines after GSK3α and GSK3α/β silencing compared to the control at the corresponding time points. ‡ = *p* < 0.05 difference between GSK3α and GSK3α/β silencing. For number of counted spines refer to Table 3. Data are presented as the mean spine width per cell ± s.e.m. The curve between time points is extrapolated. C. Spine l/w ratio changes are presented as cumulative histograms of the l/w ratio at 3 time points.

We also observed substantial GSK3-isoform specific differences in spine response to chLTD. When GSK3β was silenced, chLTD-induced spine shape changes were similar to the control, pSUPER^GFP^-transfected cells, i.e., approximately a 2% decrease in spine width maintained throughout the recovery phase. Knockdown of GSK3α and shRNA targeting both GSK3α and β restricted spine shrinkage during chLTD to a maximum reduction of 3% and enabled spines to regain their shape during the recovery phase (Fig 5A and 5B). The length of spines in the experimental variants did not differ significantly from the control condition (S1 Fig). When we monitored l/w ratio, spine structure persevered in GSK3α and GSK3α/β knockdowns (Fig 5C). In support of the specificity of GSK3α and GSK3α/β knockdown-mediated phenotypes, none of the scrambled shRNAs blocked chLTD-induced changes (S2 Fig). These results indicate that GSK3α, but not GSK3β, has a specific role in controlling spine structural changes induced by chLTD.

## Discussion

Signal transmission leaves a physical imprint on spine shape. Here we show that the simultaneous short-term inhibition of GSK3α and GSKβ hinders spine structural changes induced by mechanistic and neurotransmission-dependent triggers. However, differences in their mode of action are apparent when GSKs are blocked individually.

A change in the shape of dendritic spines involves F-actin remodeling. We have identified GSK3α and GSK3β as contributors to the regulation of this process. In a purely mechanistic LatrB model, spine shrinkage arises directly from the inhibition of actin polymerization, what shifts the balance towards F-actin depolymerization and decreases the tension of F-actin on spine head plasma membrane. Also spine shrinkage induced by LTD was considered effect of F-actin depolymerization due to cofilin activity [3]. Accordingly, one could speculate that GSK3 activity is needed for F-actin depolymerization during spine remodeling. Consequently, our results could mean that GSK3α, rather than GSK3β, is specifically needed for this process during chLTD. Yet, recent results of Halpain group show that in fact, spine shrinkage during LTD might be the result of insufficient F-actin polymerization [26]. Thus, based on our results, it is impossible to rule out that during chLTD GSK3α prevents F-actin polymerization rather than accelerates its depolymerization. However, to explain the link between GSK3 and F-actin-driven spine shape changes, several potential mechanisms must be considered because of the large variety of GSK3 effectors that indirectly and directly regulate the cytoskeleton [27,28]. Our efforts to pin-point individual proteins (e.g., p190ARhoGAP, cofilin, gephyrin and several microtubule binding proteins) behind this phenomenon did not bring an ultimate answer (i.e. we did not obtain statistically significant differences). For example, under tested conditions we did not observe substantial changes in intensity or distribution of immunostaining against phospho-cofilin (Ser 3) or distribution of gephyrin or MAP2 immunofluorescence. While our attempts do not rule out involvement of those substrates for example locally at synapses, they also suggest that in case of such important kinases like GSK3 more high-throughput approach is needed. Speculative mechanisms include: (*i*) GSK3-dependent inhibition of APC may lead to decreased activity of Rac/cdc42 [27,29] and (*ii*) GSK3-driven inhibition of p190ARhoGAP may cause activation of RhoA [30]. Both speculative mechanisms result in the formation of less branched actin and a slower full recovery of spine shape. Nevertheless, the absence of F-actin changes in chLTD can be secondary to the effects of GSK3α knockdown on substrates related to synaptic response. For example, eIF4E-dependent protein synthesis that was shown to be important for LTD-maintenance and spine morphology [31] may be a subject of GSK3-dependent regulation. Rationale behind this scenario is that in non-neuronal cells GSK3 downstream effectors include ribosomal protein S6 kinase (S6K1) and 4E-BP that can directly control eIF4E-dependent translation initiation and elongation in cap-dependent protein synthesis [32,33]. It is very challenging to define key substrates for the differential roles of GSK3α and GSK3β with currently available tools. For example, specific inhibitors of GSK3α [34] are not useful for *in vitro* culture experiments because the determination of a working concentration within the cell requires knowledge about specific GSK3α targets (that are not phosphorylated by GSK3β), which are not currently known. Moreover, GSK3α/β phosphorylate dozens of cellular targets [28]. Within a spine, each target may play a small role but the sum of their individual actions eventually leads to the changes in the local biochemistry that result in the spine shape changes. Time is an additional variable because long-term GSK3β depletion [10] reduces spine density–a phenomenon that we did not observe during the short-term inhibition of GSK3 activity. These observations are consistent, because spines that have lost their ability to respond are eliminated over time.

Further study is required on the roles of GSK3α and GSK3β in neuronal physiology, especially in different forms of synaptic plasticity, as well as in GSK3-related diseases of the nervous system (e.g., Alzheimer’s disease, bipolar disorder). Designing experiments to investigate both GSK3s will challenge the scientific community to develop kinase-specific approaches for the selective inhibition of GSK3α or GSK3β. Future studies will also require other models of synaptic plasticity, including a structural model, to determine the relative contributions of GSK3α and GSK3β.

## Supporting Information

S1 Fig Temporal spine length change. Pharmacological inhibition of GSK3α/β (A,B,C) or GSK3α/β silencing (D,E,F) do not affect fluctuations in dendritic spine morphology during 90 min. of observation (A,D), LatrB treatment (B,E) or chLTD (C,F). Data are presented as the mean spine length per cell ± s.e.m. The curve between time points is extrapolated.
(TIF)

S2 Fig Scrambled shRNAGSK3 has no effect on chLTD-induced changes in dendritic spine morphology. A. Experimental outline with 3 time points for microscopy: baseline, chLTD induction, end of recovery period. B. Representative micrographs of DIV18 cultured murine hippocampal neurons transfected with scrambled shRNA constructs as indicated. Scale bar = 2.5 μm. C. Quantitative analysis of spine shape changes; # indicates p<0.05 for measurements after GSK3α silencing compared to the control at the corresponding time point. Number of spines counted: control = 213, shGSK3α = 278, shGSK3β = 255 and shGSK3α/β = 171. Data are presented as the mean spine length or width per cell ± s.e.m. The curve between time points is extrapolated.
(TIF)
