# Supplementary figures and images for: Structural Plasticity of Dendritic Spines Requires GSK3α and GSK3β
Source: PLoS One. 2015 Jul 24;10(7):e0134018. doi: 10.1371/journal.pone.0134018 (PMC4514647; doi:10.1371/journal.pone.0134018)

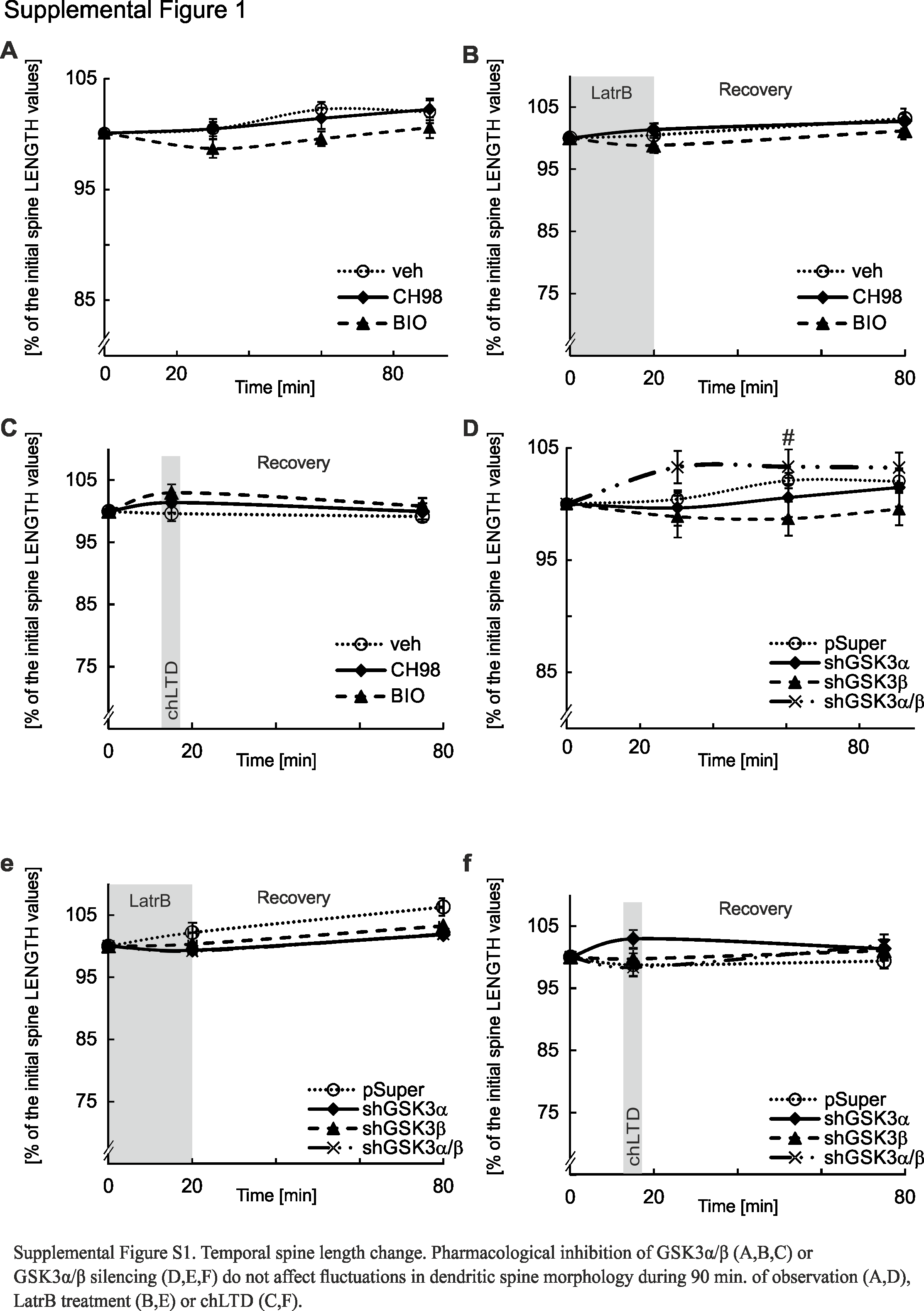

Supplement: S1 Fig — Pharmacological inhibition of GSK3α/β (A,B,C) or GSK3α/β silencing (D,E,F) do not affect fluctuations in dendritic spine morphology during 90 min. of observation (A,D), LatrB treatment (B,E) or chLTD (C,F). Data are presented as the mean spine length per cell ± s.e.m. The curve between time points is extrapolated. (TIF) [file pone.0134018.s001.tif]

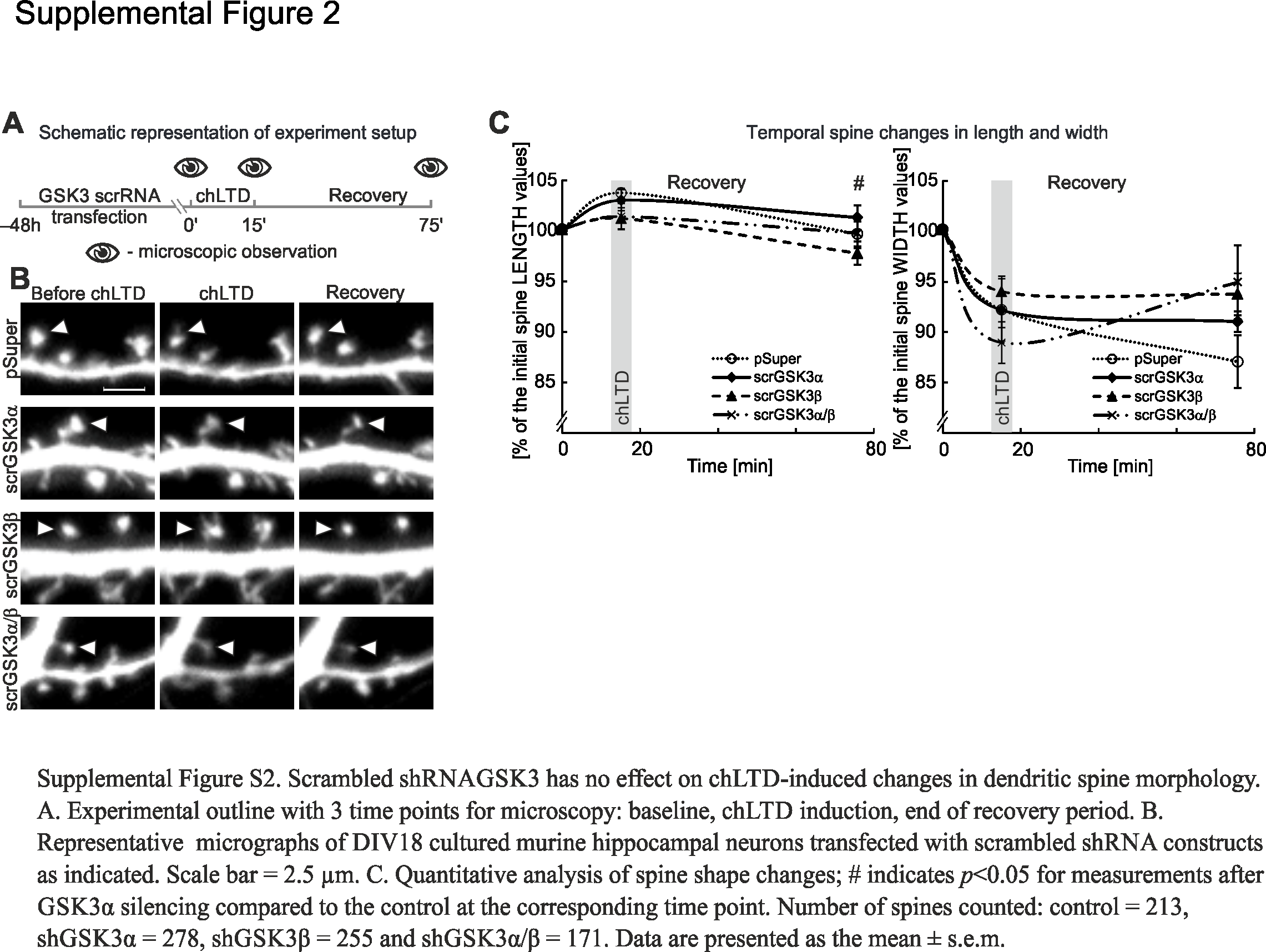

Supplement: S2 Fig — A. Experimental outline with 3 time points for microscopy: baseline, chLTD induction, end of recovery period. B. Representative micrographs of DIV18 cultured murine hippocampal neurons transfected with scrambled shRNA constructs as indicated. Scale bar = 2.5 μm. C. Quantitative analysis of spine shape changes; # indicates p<0.05 for measurements after GSK3α silencing compared to the control at the corresponding time point. Number of spines counted: control = 213, shGSK3α = 278, shGSK3β = 255 and shGSK3α/β = 171. Data are presented as the mean spine length or width per cell ± s.e.m. The curve between time points is extrapolated. (TIF) [file pone.0134018.s002.tif]
